# New Orleans school meal programs during the COVID-19 pandemic: challenges and innovations identified through qualitative interviews

**DOI:** 10.1186/s12889-024-19107-3

**Published:** 2024-06-17

**Authors:** Megan B. Knapp, Kristine Creveling, Domonique Washington, Naana Ennin, Tiffany Numa

**Affiliations:** 1 Xavier University of Louisiana, 1 Drexel Dr, 70125 New Orleans, Louisiana, USA; 2 Propeller: A Force for Social Innovation, 4035 Washington Ave, New Orleans, LA 70125 USA; 3 UNFI Foundation, 313 Iron Horse Way, Providence Rhode Island, 02908 USA

**Keywords:** COVID-19, School meals, Childhood nutrition, Food insecurity

## Abstract

**Background:**

School meal programs are critical to reducing childhood food insecurity. This study identified challenges and innovations in school meal service in a disaggregated charter school system during COVID-19 in New Orleans, Louisiana.

**Methods:**

Semi-structured qualitative key informant interviews were conducted with school officials and school food providers. Interviews were recorded, transcribed, and coded. Using an immersion-crystallization approach, patterns were identified.

**Results:**

Nine participants described challenges and solutions/innovations in food service focused around five themes: food service, procurement and costs, staffing, communication and outreach, and collaborations and partnerships. Participants faced challenges in meal service logistics, procuring food and supplies, staffing shortages, timely communication, lack of city-wide coordination, and the need to rapidly shift operations due to an evolving pandemic. While the disaggregated system created challenges in a city-wide response, the decentralized system along with policy changes offered opportunities for flexibility and innovation in meal programs through new partnership and coordination between schools and community, development of new processes for food service and procurement, and diverse modes of communication.

**Conclusion:**

These findings add to the understanding of challenges faced and innovations implemented to continue school meal programs in a disaggregated school system. Collaboration with community organizations, leveraging resources, coordinated communication, and policies allowing for flexibility were key to response and should be encouraged to build capacity and resiliency in emergencies. In future city-wide emergency preparedness planning efforts, school leaders and food providers should be included in the planning to ensure continued equitable food access for students.

**Supplementary Information:**

The online version contains supplementary material available at 10.1186/s12889-024-19107-3.

## Background

Before the COVID-19 pandemic in 2020, over half of American students relied on federally funded school meals as their primary source of nutrition with many students consuming up to half their daily calories at school [1, 2]. In New Orleans, Louisiana (NOLA), these rates were higher with 67% of students eligible to participate in the federal nutrition program [3]. After the start of the pandemic in 2020, an estimated one in three children under the age of 18 were living in food-insecure households, likely increasing the dependence on school meals [3, 4]. School meals are a critical source of nutritious food for students in food-insecure households.

As schools across the United States (US) shifted to remote learning amid fears of COVID-19 transmission in March 2020, students who relied on school meals were left in a vulnerable situation [5]. Food insecurity increased across all racial and ethnic groups; however, minoritized groups, especially Black individuals, experienced significantly higher rates of employment loss and food insecurity [6].

While COVID-19 presented unprecedented challenges, it also created an opportunity to reimagine school meal programs. To continue providing meals to students while minimizing COVID-19 exposure, the Families First Coronavirus Response Act of 2020 authorized the United States Department of Agriculture (USDA) to issue several school meal regulation waivers [7]. These waivers provided flexibility and allowed for off-site school meal consumption in non-congregate settings; flexible serving times; multiple meals served at one time; meals to be picked up without a student present; eased nutritional requirements; meals to be served at no cost to all students in an area, regardless of income; and increases in reimbursement rates [7, 8]. In 2022, the Keep Kids Fed Act extended partial school meal flexibilities through the 2022–2023 school year and included relaxed nutritional standards, increased reimbursement rates, and free meals for eligible students but discontinued the waiver to provide free meals for all students [7]. Many states, including Louisiana, also provided pandemic electronic benefits transfer (P-EBT) through the summer of 2022 to eligible families to purchase food during school closures [9].

Unlike many cities with traditional public schools operated by a school district, the NOLA school system consists of almost entirely of charter schools, which are publicly funded schools that are autonomously operated by decentralized nonprofit community boards who set their own policies and regulations. Differing from traditional public schools, all student enrollment in New Orleans is based on school choice and not residency [10]. The system of disaggregated school governances and student resident proximity to schools required additional efforts and coordination to ensure students could access meals. While several studies have identified challenges and innovations in school meal service during COVID-19 school disruptions, this study focused on the response in a city-wide charter school system with decentralized leadership, which poses unique challenges for coordinated response in emergencies [11–15]. Findings from this study can inform future policies, practices, and emergency preparedness plans to improve equitable food access and reduce food insecurity among students, especially for those within disaggregated school systems.

## Methods

Using a qualitative approach, semi-structured key informant interviews were conducted in May 2022 with school officials and food service management companies involved in COVID-19 school meal operations in NOLA.

With guidance from the Getting to Equity framework and previous school-based meal research, open-ended, semi-structured interview questions were developed by the research team [11–14]. The questions were reviewed and pilot tested by Louisiana Department of Education and Healthy School Food Collaborative staff. The questions focused on six topics: changes in food service; facilitators; barriers; student and family need; equity; and partnerships (Supplement 1). A short paper-based survey collected basic interviewee demographic characteristics and school information including types and number of schools served and student population size.

Interview participants were selected through purposive sampling strategies based on their experience and knowledge of school meal operations [16]. With assistance from a local food service operations collaborative, the research team identified 18 school administrators and 2 food service management companies that were involved in the food operations of the 27 school networks that serve NOLA students. Of these individuals, nine had been employed in school food operations during school closures between March and August 2020 and were able to provide interviews on the experience. Contact information for the nine eligible individuals was attained through existing community connections, and recruitment emails were sent out by the research team.

After agreeing to participation and providing written consent, participants completed the demographic characteristics and school information survey. All nine individuals participated in the surveys and interviews. Seven were school leaders involved in food service and two were food service management company directors. Six participants identified as male and three as females. Most (*n* = 6) participants identified as white, two identified as black/non-Hispanic, and one identified as Hispanic. All nine participants worked with charter school networks that served elementary and middle schools. Eight of the participants also served early care and education programs, and five served high schools. Participants represented or served a range of 3 to 70 schools with student populations of 1,400 to 45,000.

One team member, a female, non-academic community partner with a Masters in Public Health and Registered Dietitian credentials, conducted all 1-hour interviews via an online meeting platform, Zoom. Zoom was chosen for its audio recording and transcript generation ability. The interviewer used a private internet network and conducted the interviews in a private space. To ensure confidentiality, participants were sent a direct link, and virtual waiting rooms were set up to restrict access to the online meeting space. All interviews were audio-recorded and transcribed using the online meeting platform functionality. Participants were offered a $30 gift card for their time.

### Data analysis

The transcripts were analyzed using thematic analysis with an immersion-crystallization approach consistent with similar research [12, 17]. The interviewer reviewed the transcripts and recorded their initial thoughts after each interview. Transcripts and notes were then reviewed by two research assistants for accuracy and transferred into NVivo version 13, a qualitative research software used to organize and code the data [18, 19]. Three team members, which included a behavioral scientist with expertise in qualitative research and two student research assistants, independently reviewed each transcript and developed an initial set of codes based on patterns in the transcripts by questions and then across questions. The three coders met regularly to discuss and compare coding and discrepancies to ensure reliability. After coding was complete, the team worked together to identify and finalize themes. The themes were reviewed and validated by a fourth team member who conducted the interviews. All team members agreed on the final codes, and quotes were selected to illustrate themes. After the coding was complete and themes were finalized, a brief was created and distributed to all interviewees for feedback and to confirm findings.

## Results

Five themes were generated from the analysis process: food service, procurement and costs, staffing, communication and outreach, and collaborations and partnerships. Within each theme, subthemes were identified and grouped by challenges and solutions/facilitators/innovations.

### Food service

#### Challenges

In general, participants noted that food service models varied among schools and across time as the pandemic guidelines evolved and as USDA and state-level waivers were issued. Most participants discussed challenges in the need to shift service style quickly throughout the pandemic. Little time was given to adjust plans, and schools were unsure how many students needed meals and how to logistically distribute meals to students while ensuring safety and quality. Highlighting this struggle and time of uncertainty, one participant said, *“How do we deliver meals? I was googling trying to figure it out.”*

##### Distribution difficulties

Within Orleans Parish, students may not live near their schools, which complicated distribution. Some families did not have reliable transportation to school distribution sites or had working schedules incompatible with distribution times or locations. One participant stated, “*The biggest challenge was not making the food but distributing it.”* Some schools began with daily drive thru sites and eventually moved to delivery services to better meet student needs. Other networks did not have the capacity to offer delivery and struggled with verifying addresses for students, scheduling deliveries, and hiring drivers.

After schools re-opened or offered hybrid learning, participants reported that schools served meals in classroom pods and provided deliveries for those quarantined or participating in online instruction. With classroom deliveries, some schools lacked carts for transporting food, experienced difficulty in preparing portable plates, and produced large amounts of environmental waste. The additional staff time to prepare and transport food and increased cleaning costs were also barriers.

##### Food safety and quality

With supply chain issues and shifting delivery models, schools also faced challenges with food safety and quality. As detailed by a participant, *“It was difficult in the beginning to prepare food, box it in a way that we had never done before, keep food temperatures safe, and then distribute it outside of our building.”* Decreases in produce and increases in prepackaged and processed food with lower nutritional quality and higher sugar content for delivered meals were noted.

##### Changes in participation

Participants observed a drop off in school meal program participation across the city in late summer. Theories for the decline included opening of other community feeding sites, P-EBT availability, limited menu options, and menu fatigue. *“There was an element of people getting tired of eating the same food meal after meal, day after day. The variety that we were not able to do was a challenge.”* One participant believed that having families complete pick up forms required by the state were a deterrent to participation.

#### Solutions/Innovations/Facilitators

##### USDA waivers flexibility

During school closures, schools took advantage of the USDA waiver allowing non-congregate feeding and offered community and school drive thru and grab-and-go sites, door-to-door deliveries, or a combination of both. Participants recognized that USDA waivers were key in continuing school meal programs and allowed for flexibility in food distribution strategies to improve food access and increase participation. As highlighted by a participant, *“USDA waivers were so successful in making sure children had access to food. […] Things get challenging, but that flexibility to be creative and innovative is helpful.”*

##### Collaboration/Partnerships for equitable distribution

Once waivers allowed schools to serve students in all areas, school networks collaborated to make sure access was equitable across the city, and families could pick up meals from any site. *“We started to track areas of the city that maybe needed an additional site opened, and we would work together to figure out which school could manage it. When everybody has food for their neighborhood children, it works better. Wherever you are in the city, you can go somewhere and get food.”*

This collaboration led to the creation of interactive maps for families to find their nearest distribution sites. Sites were chosen based on the ability to serve large populations within walking distance and included schools, city recreational centers, churches, business and shopping mall parking lots, and public transportation hubs and stops. Some schools partnered with community organizations to pick up meals for students in their neighborhoods and under their care. Rideshare companies distributed free codes to transport families on meal distribution days.

One participant noted their school networks offered door-to-door deliveries from the start of school closures to address transportation and other access barriers. To help with deliveries, schools hired out of work bus drivers and engaged in partnerships with established local food delivery programs and companies with refrigerated trucks. One participant expressed that participation in meal programs was at its highest with delivery, *“Students were saying how much they felt appreciated, love, support, and fed because we were delivering food straight to their house. Many of them have no food at home. There was literally no lift from their side.”*

Highlighting the extent of collaborative efforts to distribute food to students in most need, one participant noted, *“We did grocery shopping, we pulled stuff out of our school gardens. We did delivery to people’s houses with fresh food. There were restaurants donating 100 meals a day.”* This creativity in distribution and partnerships was critical to continued meal service.

##### New offerings and packaging

To address food service challenges, schools offered a variety of new offerings and packaging including options such as hot meals, daily meal bags, five-day meal kits, and family pantry kits of shelf stable items with many schools taking advantage of waivers allowing for distribution of batch meals. To ensure food safety and quality, schools provided flyers with nutritional information and instructions for storing, reheating, and expiration. Operations with the ability to heat seal meals were able to provide more food options with higher nutritional quality and avoided reliance on prepackaged foods. Two participants remarked that the ability to purchase and store more shelf stable foods for emergencies would be helpful in the future.

### Procurement and costs

#### Challenges

##### Shortages

Procurement of food and supplies was a common challenge cited by interviewees. As described by one participant, *“We purchased things we never bought before, and it was hit or miss with what we could and couldn’t source.”* Shortages in foods sourced from aggregated supply chains, such as grains, cereals, milk, chicken, and burgers, were noted. Schools purchasing Department of Defense produce described shortages and less variety in produce options. Participants also mentioned difficulty in obtaining supplies such as personal protective equipment (PPE), sanitizer, thermometers, physical barriers to separate staff, packaging for food, food warmers, insulated bags, and food carts for transporting food. Further, pre-pandemic contracts were in place for products and services that could not be fulfilled.

##### Rising costs

While participants noted feeding students, not controlling costs, was the main concern at the time, finances were a challenge, specifically rising costs of food and purchasing of packaging, food storage solutions and equipment, and PPE. Due to the disaggregated charter school system, schools were often on their own for purchasing and unable to take advantage of economies of scale and group purchasing power. In addition, schools noted a need to pay staff more for retainment and recruitment due to availability of stimulus money and unemployment funds.

##### Attendance and reimbursement

In the shifting education delivery models and quarantines, some schools struggled with attendance and securing reimbursement for meals without students to consume them. Estimating meal quantities for each week was a costly challenge. One interviewee said, *“The inventory is interesting, because you can’t forecast if you don’t know what classrooms are going to be quarantining […] There was a lot of waste, and our food costs were way higher. We lost a lot of money on food.”*

#### Solutions/Innovations/Facilitators

##### Flexibility of USDA waivers and other policy changes

Several participants discussed how the USDA waivers on nutritional requirements helped them overcome procurement challenges and expand offerings. *“When you’re given a waiver, you’re struggling to get grains, and you’re trying to pack and serve things that need to last a week, [the USDA waivers] made it just easy.”* Higher reimbursement rates with waivers gave schools flexibility to pay higher prices, amend contracts, engage new vendors, and reduce debt. In addition, the governor’s emergency declaration was recognized as a facilitator that allowed schools to procure food from new vendors. Turning to local producers and suppliers was one solution school networks employed to address supply chain issues and shortages.

##### Grants, cost-sharing, and budget reallocations

Three participants noted they were able to receive additional grants to help cover costs of food services staff, food, deliveries, and equipment. Further, some participants found ways to work with other school networks to share costs and delivery routes. Re-allocation of budget line items also resolved some budgetary challenges. As noted by one participant, *“We could use bus money to help support food and [delivery] service.”*

### Staffing

#### Challenges

##### Morbidity and mortality

Participants noted several challenges in staffing for their meal operations. COVID-19 morbidity and mortality were high among staff and staff family members, and many food service staff were at high risk for complication due to age. Protocols for protecting staff were unclear, and retention was difficult due to illness, quarantines, and fear.

##### Need for more staff

Compounding the issue, school meal operations had smaller teams but more staffing needs. *“You’re taking extra steps in terms of packaging food; moving it from point A to point B. We actually needed larger teams, and we had smaller teams everywhere.”*

#### Solutions/Innovations/Facilitators

##### Flexibility

Flexibility in staff scheduling, pay, roles, and hiring was key to maintaining operations. Several participants noted they were invested in keeping food service workers employed. One interviewee explained, “*We want to keep people who are committed to our kids. We want the people who have relationships with their kids, who care about their work.”* Some schools worked with employees on flexible schedules and increased pay as incentives. For schools experience staffing shortages, solutions included hiring restaurant staff, relying on community volunteers, and shifting roles of staff to help with logistics. *“We were heavily using leadership teams and staff members who were willing to come in and help.”*

### Communication and outreach

#### Challenges

##### Unclear and late communication

As USDA waivers were issued, participants noted minimal federal communication and guidance. Policy changes and decisions around school operations were often last minute, and schools were left with little time to plan and communicate effectively with parents about meal distribution. One participant commented that interpretation varied by school networks, leading to initial differences in responses across the city, divergent messaging, and confusion for parents. Different networks were communicating different information to parents, and greater city-wide coordination was needed. In addition, some schools were not equipped to communicate in all languages of their students.

#### Solutions/Innovations/Facilitators

##### Collaborative, unified messaging

Collaboration among schools that may otherwise be competitors was key in effective response. Once USDA waivers were in place to allow schools to feed all students in their community, networks began to work together with lawyers to understand the policy changes and waivers and create a unified, simple strategy and message. *“Our messaging became very much about all NOLA schools as opposed to individual organization. That might actually be the first time that ever happened in our charter system.”*

##### Diverse communication strategies

Participants reported using a wide range of communication strategies and platforms to increase timely messages to families about meal availability. The methods used included: social media, robo-calls, text messaging, school websites, email, neighborhood banners, and car flyers. Online platforms and 1-800 numbers were used for delivery service registration. Several participants commented that the most effective way to communicate messages was teacher outreach to families and using social workers. One participant noted that teachers were especially helpful in reaching undocumented guardians who may have been hesitant to register. Parent surveys were utilized to capture family needs and create two-way communication with parents. To ensure accessibility in communication, the city government assisted with translation services.

### Collaborations and partnerships

#### Challenges

##### Disaggregated system

As noted above, NOLA charter schools had not collaborated often prior to the pandemic, and the disaggregated governance system of charter schools led to differences in response and communication strategies. Further, participants noted challenges in understanding which schools and vendors were able to procure supplies and resources.

#### Solutions/Innovations/Facilitators

##### Interschool collaboration

While disaggregated governance was a challenge, several participants stated that the variance in charter school structures and governance also led to greater flexibility and creativity. “*We could be really innovative with how we used the benefits of each kind of structure as opposed to the barriers of every structure […] We used our collective flexibilities and then combined them to create a safety net.”* Through this time, a framework for charter school collaboration was developed. As describe by a participant, *“If we ever have to step into the space again, we definitely have a blueprint of how to start.”*

Charter schools partnered and held daily phone calls to ensure consistency in resource accessibility for families. *“It was the first time that all charters in the city worked together on a real thing that served our city as a whole […] We all agreed to follow the same set of policies to make it easier on the city and easier on families.”* One school network took a voluntary lead role in convening school leaders and provided safety guides and PPE for kitchen staff.

##### External partnerships

The city assembled work groups that included the health department, early childcare, and other stakeholders to discuss barriers, assess supplies, share best practices, and coordinate transport of food. Schools also partnered with the city to create a tracking system to determine meal availability. City-wide coordinated efforts ensured families in need were directed to an access point.

Working with food service management companies and nonprofit organizations with clients in other states also helped with innovation and planning. One participant commented that without the nationwide view, *“I don’t think we would have been able to feed kids nearly as fast or as healthy.”* At the local level, school participation in the city’s emergency operations and tabletop exercises was recognized as key to preparation and action.

Participants noted collaboration with food banks, mutual aid organizations, philanthropists, and health services at food distribution sites. *“Our food sites became one of the easiest ways for the philanthropic community to engage with families.”* Sites provided school supplies and technology, toiletries, diapers, formula, and art supplies. To mitigate food waste, schools partnered with food access organizations to distribute surplus meals to communities. External partnerships expanded organizational capacity and reach of services and resources.

## Discussion

As schools closed to curb the spread of the COVID-19 virus, New Orleans school networks faced many challenges, and innovation was critical in continuing school meal programs for children. Like other cities, the uncertainty and evolving guidelines complicated food distribution, communications, and policy implementation; rising costs, supply chain disruptions, and unpredictable program participation led to procurement and budgetary issues; and illness and efforts to prevent spread strained staff and increased staffing needs [11–15, 20]. As the NOLA public school system consists of almost all charter schools with their own governances, schools and food service management companies faced an additional layer of coordination challenges between school networks as networks were competitors and not accustomed to working together. While the disaggregated system created some barriers in city-wide response, the decentralized system and USDA waivers offered opportunities for flexibility and innovation in meal programs through new partnership and coordination between schools and community, development of new processes for food service and procurement, and use of diverse communication strategies and channels. Outside of partnerships with other school networks, collaboration with community and business partners helped schools to leverage resources and funds, understand food availability, and mitigate food insecurity in this as well as other studies [11, 14, 15]. The efforts required for continuation of school meal programs led to a blueprint and insight for future disaster preparedness among a city-wide charter school system.

The flexibility allowed by USDA waivers was recognized as a key facilitator to continuation of school meal programs and encouraged innovation in food distribution while minimizing the risk of transmitting COVID-19 [8, 11–14]. Like other studies, meal service logistics, food and supply procurement, transportation, understanding waivers, staffing, and the need to rapidly shift operations were challenges for school networks and food service management companies [11–13]. Using waivers, schools were able to implement a variety of food service models, work with new vendors, purchase new products, offset increased costs, and engage in partnerships to address barriers and ensure equitable access to food [20, 21]. While program participation was unpredictable at times, emerging research has shown that USDA waivers and universal school meals increased participation in school meal programs, and policymakers should consider universal school meals as a permanent policy to overcome the impacts of the pandemic on students and their families and address food insecurity [15, 22].

The importance of interschool as well as cross-sector collaboration and partnerships in food distribution and communication was highlighted throughout the interviews in New Orleans as well as in research in other cities [11, 12, 15, 23]. These collaborations may have led to the ability to serve students in socially vulnerable neighborhoods during the COVID-19 school closures in New Orleans [21]. Future disaster planning may consider focusing on strengthening relationships and communications between schools and between external community partners while ensuring that schools have a voice in citywide planning [24]. In addition, city governments or school networks may consider creating a role for a city-wide food coordinator or further support and engage with local food policy councils, such as New Orleans Food Policy Action Council, to facilitate collaborations [25]. This role or council could convene school leaders, businesses, and organizations to aid in city-wide communication, partnerships, group purchasing, translation services, meal distribution, and supply inventory for coordinated action and expanded community capacity [26].

As highly aggregated supply chains in school meal operations led to shortages and disruptions for key food items, investment in local supply chains and policies that support local food systems may optimize resiliency in future crises and reduce reliance on national or global supply chains [15, 27, 28]. Additionally, schools may consider investing in heat seal technology, which allowed for preparation of greater variety and nutritional foods options, and stockpiling of shelf stable items that could be distributed in case of emergencies to bolster capacity to meet student meal needs.

Finally, while the waivers and policy changes did allow for responsiveness and flexibility, lack of clear, timely guidance may have hindered efficiency of response [15, 24]. In order to help schools mobilize quickly in times of disruption, future efforts should ensure clear and quick guidance and technical assistance on policy changes at the local, state, and federal level [23, 24].

### Strengths and limitations

This study gained in-depth perspectives from both school leaders and food service management companies that played pivotal roles in school meal programs during school closures. The study also provided context for understanding how schools with disaggregated governance were able to coordinate and serve students in need during COVID-19 [21].

In recruiting participants for this study, several individuals who had been in leadership and decision-making roles during the pandemic no longer worked with their organizations, and this limited the ability to interview all stakeholders and potentially leading to selection bias. Those that had left their positions may have different perspectives. In addition, this study did not include viewpoints from the user end (families), which may be helpful for deeper understanding. Further, as the NOLA charter school system is unique, some results may not be generalizable to other cities.

## Conclusions

Overall, this study adds to the understanding of challenges faced and facilitators, solutions, and innovations implemented to continue school meal programs in a disaggregated charter school system. School meal programs are critical for food security and well-being of students, and school leaders and food service management companies should be included in future emergency preparedness planning. Collaboration with community organizations, leveraging resources across school networks, coordinated communication, and policies allowing for flexibility were key to response and should be encouraged to build capacity and resilience in disasters. These findings have been shared with NOLA schools, food service management companies, and relevant city agencies for planning for future crises.

### Electronic supplementary material

Below is the link to the electronic supplementary material.

Supplementary Material 1

## Data Availability

The data used and analyzed during the current study are available from the corresponding author on reasonable request.

## Abbreviations

NOLA: New Orleans, Louisiana
P-EBT: Pandemic Electronic Benefits Transfer
US: United States
USDA: United States Department of Agriculture
