# Supplementary material for: New Orleans school meal programs during the COVID-19 pandemic: challenges and innovations identified through qualitative interviews
Source: BMC Public Health. 2024 Jun 17;24:1614. doi: 10.1186/s12889-024-19107-3 (PMC11181593; doi:10.1186/s12889-024-19107-3)
Supplement: Supplementary file 1 — Supplementary Material 1 [file 12889_2024_19107_MOESM1_ESM.docx]

**Supplement 1: Qualitative Interview Guide**

As you are aware, in collaboration with researchers at BLINDED, we are conducting a study to increase our understanding of pandemic-related school feeding response in Orleans Parish by conducting key informant interviews of ten school leaders and other food access stakeholders. Findings will inform policies and practices to improve food access and health equity for children of New Orleans. You have been asked to participate due to your knowledge and involvement with school feeding programs. We appreciate your participation.

In today’s interview, we are going to ask questions around 6 topics: changes in food service; facilitators; barriers; student and family need; equity; and partnerships.

**Changes in Food Service Operations**

To get us started, please describe changes made to how you procured, prepared, and served school meals in spring 2020, 2021, and now.

**Challenges/Barriers,**

Now, let’s turn to challenges of changes to school meal service. Please share challenges or barriers your schools have faced during the pandemic.

- Describe any different or unique challenges or barriers during remote learning.
- And now, with schools reopened, do you have any different or unique challenges or barriers?

**Facilitators, Insights, and Future Directions**

- Please tell me more about any changes that supported your schools’ capacity to serve meals during school closures and reopening. Are there any best practices or lessons learned you would want to carry forward for future school closures or in a normal school meal service operation?

**Student and Family Needs**

Shifting to think about student and family needs,

- Since the spring closures, what are the key gaps in meeting student and community food needs? How has your district tried to address any of these needs? How did student or family input shape your reopening meal service approach?

**Equity**

- What strategies were implemented to ensure equitable accessibility to meals?  How were meal distribution sites chosen?
- What communication strategies were used to notify parents of meal availability, eligibility, and distribution plans?  What strategies were effective and ineffective?  What strategies would you employ in the future?

**Partnerships**

- Now, let’s talk about key partners. What individuals or groups were consulted when your district made changes to food service operations during school closures and then upon reopening?
- What community resources, if any, were leveraged to increase meal availability and/or accessibility for students and families?

We appreciate your thoughts and insights and thank you for your time and participation. We will be in touch once we have compiled our findings.
